# Editorial: The Schistosomiasis Vaccine – It is Time to Stand up

**DOI:** 10.3389/fimmu.2015.00390

**Published:** 2015-07-30

**Authors:** Rashika El Ridi, Ahmad A. Othman, Donald P. McManus

**Affiliations:** ^1^Zoology Department, Faculty of Science, Cairo University, Cairo, Egypt; ^2^Medical Parasitology Department, Faculty of Medicine, Tanta University, Tanta, Egypt; ^3^QIMR Berghofer Medical Research Institute, Brisbane, QLD, Australia

**Keywords:** *Schistosoma mansoni*, *Schistosoma haematobium*, schistosomiasis vaccine, paramyosin, Sm14, schistosome peptidases, type 1 and type 2 immunity

Schistosomiasis is a severe parasitic disease, endemic in 74 developing countries with up to 600 million people infected and 800 million, mostly children, at risk of contracting the disease following infection predominantly with *Schistosoma mansoni*, *Schistosoma haematobium*, or *Schistosoma japonicum*. The disease burden is estimated to exceed 70 million disability-adjusted life-years, and leads to remarkably high YLD (years lived with disability) rates. Even more importantly, people with schistosomiasis are highly susceptible to malaria, tuberculosis, and hepatic and acquired immunodeficiency viruses. There is only one drug, praziquantel, currently available for treatment and it has high efficacy, low cost, and limited side effects. However, only 13% of the target population has received the drug, and those treated are at continuous risk of reinfection necessitating repeated drug administration and the emergence of drug-resistant parasites is a constant threat (1). Currently there is no vaccine. The *a priori* requirements for discovery of a vaccine formulation include the following: identification of protective key immune players in humans; characterization and isolation of target antigens; establishment of efficacy in terms of reduction of parasite burden as well as amelioration of immunopathology; establishment of safety; and finally, provision of considerable funds along with physical infrastructure and qualified personnel to carry out clinical trials.

The target of >40% protection has been achieved with some schistosome molecules such as fatty acid binding protein (Sm14), paramyosin, calpain large subunit (Sm80), superoxide dismutase (SOD), glutathione S-transferase (GST), glyceraldehyde 3-phosphate dehydrogenase, and cysteine peptidases (2). Furthermore, Pearson et al. (3) identified the antigens selectively recognized by serum IgG1 and IgE of *S. haematobium* patients who acquired praziquantel-induced resistance (DIR) to the infection, or self-cured macaques following *S. japonicum* infection. The probed antigens were derived from *S*. *mansoni* and *S. japonicum*, likely because of the documented antigen conservation among the three main clinically important species, and were selected among those known to be secreted or localized to the tegument. The tegument is at the host–parasite interface, but its access by host effector antibodies is entirely prevented in healthy schistosomes, otherwise they would not survive a day, not to mention decades, in the host bloodstream. Anyhow, the study identified once again calpain, SOD, and GST as vaccine candidates together with surface membrane-associated antigens such as tetraspanins and glucose transporters, as well as an array of newly discovered target antigens. A remarkable finding in the study was the implication that type 2 (IgG1 and IgE) and not type 1-related antibodies are critical for human resistance against *S. haematobium* reinfection. Besides the worm tegument, which may not be accessed by host effector antibodies, the digestive tract is the other major interface between host and parasite. Schistosome peptidases responsible for digesting blood-born cells, components, and nutrients may be targeted, and possibly neutralized and blocked, by host antibodies and, thus, represent potential vaccine candidates. The timely study of Figueiredo et al. (4) reviewed what is known about the properties and vaccine potential of proteins secreted by the esophagus, and the lining (gastrodermis) of the blind-ended gut, namely Sm14, Sm10.3, venom allergen-like (VAL) protein, Cu–Zn SOD, cathepsin B, and cathepsin L.

It is reassuring we have convened on a handful of promising vaccine candidates and several reviews in this issue illustrate the advances that have been made. Kurtis et al. (5) reviewed the discovery, gene cloning, and expression of paramyosin; its localization in muscles, just below the tegument, and in the gut lining of adult worms; its protective potential in rodents against *S. mansoni* (24–53% protection without adjuvant, associated with induction of interferon-gamma, IFN-γ) and against *S. japonicum* (62–86% protection without adjuvant); its immunogenicity in humans, whereby *S. japonicum* paramyosin was found to be the target of protective type 2-biased cytokine and antibody responses; and plans to move it toward phase I clinical trials. The history of the discovery, gene cloning and expression trials, vaccine potential, and outcomes of completed phase I clinical trials were reported for the fatty acid binding protein, Sm14, by Tendler et al. (6). Cost-effective, large-scale production of recombinant Sm14 expressed in *Pichia pastoris* is currently in place, and the protein will be formulated with glucopyranosyl lipid adjuvant-stable emulsion (GLA-SE) adjuvant. This synthetic adjuvant has been selected as it enhances type 1, namely IFN-γ, responses, identified as the basis of the Sm14-mediated protective immunity in animal models and humans. The protective potential of other prominent vaccine candidates, the antioxidant enzymes Cu–Zn SOD and glutathione S peroxidase formulated as plasmid cDNA and recombinant protein preparations, has been assessed in the Olive Baboon (7). The vaccine formulations were entirely safe and strongly immunogenic but, in accord with a plethora of previous vaccine trials involving type 1 immune response-inducing adjuvants or plasmid cDNA constructs, induced limited and/or variable protection in non-human primates against *S. mansoni* challenge infection.

Despite the fact that protective immunity to *S. mansoni* and *S. haematobium* infection in humans is documented to be dependent on type 2 immune responses (2, 3, 5, and references therein), formulations of schistosomiasis vaccines destined for use in humans still aim to induce predominant type 1-related cytokines and antibodies, clearly indicating we have not yet reached a consensus regarding the type of immune responses an anti-schistosomiasis vaccine should elicit. The review by Fonseca et al. (8) is, thus, particularly well timed as it seeks to find the optimal immune weapons generating vaccination-mediated resistance against schistosome infection via identifying the immune responses associated with protective immunity elicited by several vaccine candidates namely GST, Sm14, calpain (Sm80), tetraspanins, and Sm29 in mono- and multivalent formulations. The review emphasized and documented the importance of specific antibodies and strong IFN-γ production in parasite elimination regardless of the vaccine candidate used.

Since currently available vaccine candidate formulations mediate type 1-biased protective immunity, which is limited or partial at best, it is important to revisit the lessons of the radiation-attenuated (RA) cercarial vaccine (9). In this respect, a meta-analysis of the experimental studies undertaken with the RA cercarial vaccine in mice (755 observations from a total of 105 articles) was performed by Fukushige et al. (10), who reported that the RA vaccine has the potential to induce protection as high as 78% with a single dose of vaccine. While major predictors of protection were the immunizing cercarial number (antigen dose) and interval between the last vaccination and challenge (duration of immune memory), the study emphasized the importance of host immunization with more than a single schistosome molecule in order to achieve protection. The early pioneers studying schistosome biology helped devise an efficacious schistosomiasis vaccine by demonstrating that the physiological and reproductive status of *S. mansoni* is strongly influenced by the microenvironment of the host and that the lung and liver are the sites of innate and acquired immunity-mediated parasite attrition in permissive (mice, hamsters) and non-permissive (rats) hosts (11).

To compile a road map for the successful development of a schistosomiasis vaccine: (1) It appears we have at hand a plethora of well-characterized, ready for use vaccine candidates (2–11). (2) As noted by Fonseca et al. (8), 24 h and older schistosomula are refractory to killing by antibody-dependent complement-mediated attrition, and this fully applies to antibody-dependent cell-mediated cytotoxicity (ADCC) as well. (3) Specific antibodies may access the worm gut lumen and those that escape immediate digestion might be able to neutralize and interfere with enzymes critical for worm feeding and fecundity, but not survival, as these processes by definition impact on juvenile and adult worms not schistosomula migrating in the lung capillaries and liver sinusoids (4). (4) We are left then with the hunt and chase theory, whereby immune antibodies and cells interact with excreted–secreted parasite products in the vicinity of migrating schistosomula, alarming and activating effector immune cells (2, 9, 12). (5) Eosinophils and basophils would be particularly effective immune cells but need a type 2 immune environment for recruitment and activation (2, 9, 12, 13). (6) Protective immunity against reinfection with *S. mansoni* and *S. haematobium* in humans is documented to be associated with type 2 responses (2, 3, 5, 8). (7) There is considerable evidence demonstrating that immunization of outbred, akin to man, mice with selected vaccine candidates in conjunction with type 2 immune response-inducing cysteine peptidase, papain, or cytokines (namely interleukin-25, interleukin-33, or thymic stromal lymphopoietin) can elicit a reduction in *S. mansoni* worm burdens consistently higher than 50% and reaching the 78% level achieved by vaccination with the RA cercarial vaccine (14). (8) These molecules inducing type 2 immunity were replaced by *S*. *mansoni* cysteine peptidases, leading to consistent and highly significant (*P* < 0.0001) 50–83% protection of outbred mice against *S. mansoni* challenge infection (15). (9) It has been demonstrated that this approach, incorporating a cysteine peptidase-based vaccine, is effective in protecting hamsters and mice against *S. haematobium* as well (16). (10) A consensus should be reached without delay in order that independent, collaborative experiments could be devised and undertaken that would result in the development of a near sterilizing protective immunity-inducing schistosomiasis vaccine (2, 9).

In conclusion, discovery of a successful vaccine for a host as complex as man against a parasite as complex as *Schistosoma* is a monumental scientific challenge with many factors at play including parasite strain; intensity, duration, and frequency of infection; genetic make-up and immunological status of the host; perinatal sensitization; host nutritional status; and co-infections with other infectious pathogens. Insights of protective immune responses generated by vaccination have been deduced from experiments with rodents or, more importantly, non-human primates, but data and experience with humans are still much needed. Important considerations such as vaccine efficacy, safety, and cost, all count in the development of a successful human vaccine. It is highly unlikely that the vaccine, when available, would stand alone, but it could be a major element in an integrated control package. A primary goal should be the vaccination of children in endemic regions at an age as early as possible on the path to the elimination of schistosomiasis.

## Conflict of Interest Statement

The authors declare that the research was conducted in the absence of any commercial or financial relationships that could be construed as a potential conflict of interest.
